# Amyloid precursor protein is a subunit of microglial Hv1 channels

**DOI:** 10.1016/j.coi.2026.102751

**Published:** 2026-03-03

**Authors:** Ruiming Zhao, Steve AN Goldstein

**Affiliations:** Departments of Pediatrics, Physiology & Biophysics, and Pharmaceutical Sciences, Susan and Henry Samueli College of Health Sciences, University of California, Irvine, CA 92697, USA

## Abstract

Voltage-gated proton channels (Hv1) are key regulators of microglial activation, coupling proton extrusion to reactive oxygen species production, cellular pH homeostasis, and pro-inflammatory signaling. Dysregulated Hv1 activity exacerbates neuroinflammation and contributes to a range of central nervous system pathologies. Our recent work shows that proton channels in microglia are formed by the co-assembly of Hv1 pore-forming subunits and amyloid precursor protein (APP). APP, and its C99 transmembrane fragment, assemble with Hv1 to enhance channel activity, altering gating kinetics, modifying pharmacological properties, and amplifying inflammatory mediator release from microglia. Importantly, Alzheimer’s disease-associated APP mutations further potentiate Hv1 activity, providing a mechanistic link between genetic risk factors and microglial dysfunction, offering APP-Hv1 as a new therapeutic target for neuroinflammatory disease. This review summarizes current views of microglial Hv1 function and highlights that Hv1, long thought to operate as homodimers despite exhibiting varied attributes in native cells, exhibits functional diversity through accessory subunit incorporation.

## Introduction

The human voltage-gated proton channel (Hv1) is the membrane protein that mediates proton extrusion from cells, thereby regulating intracellular pH and membrane potential [1–3]. Hv1 is widely expressed in both innate and adaptive immune cells, where it prevents detri-mental intracellular acidification and counteracts depolarization during cellular activation [3,4]. In phagocytes such as neutrophils and microglia, Hv1 supports the respiratory burst by providing charge compensation and maintaining pH homeostasis for NADPH oxidase (NOX), thus enabling sustained production of reactive oxygen species (ROS) required for effective pathogen clearance [5–7]. Conversely, excessive Hv1 activity can promote inflammatory responses and contribute to tissue damage. Consistent with these roles, our studies have demonstrated that the specific Hv1 peptide inhibitor (C6) suppresses neutrophil migration into the lungs as well as neutrophil-derived ROS and cytokine release, thereby subduing acute inflammatory and infectious lung injury [8–10]. C6 peptide similarly attenuates microglial ROS and cytokine production [7]. In this review, we focus on Hv1 channels in microglia and their newly identified regulation by the amyloid precursor protein (APP) and C99 as accessory proteins.

## Hv1 is a key regulator of diverse microglial functions

**Figure 1:**
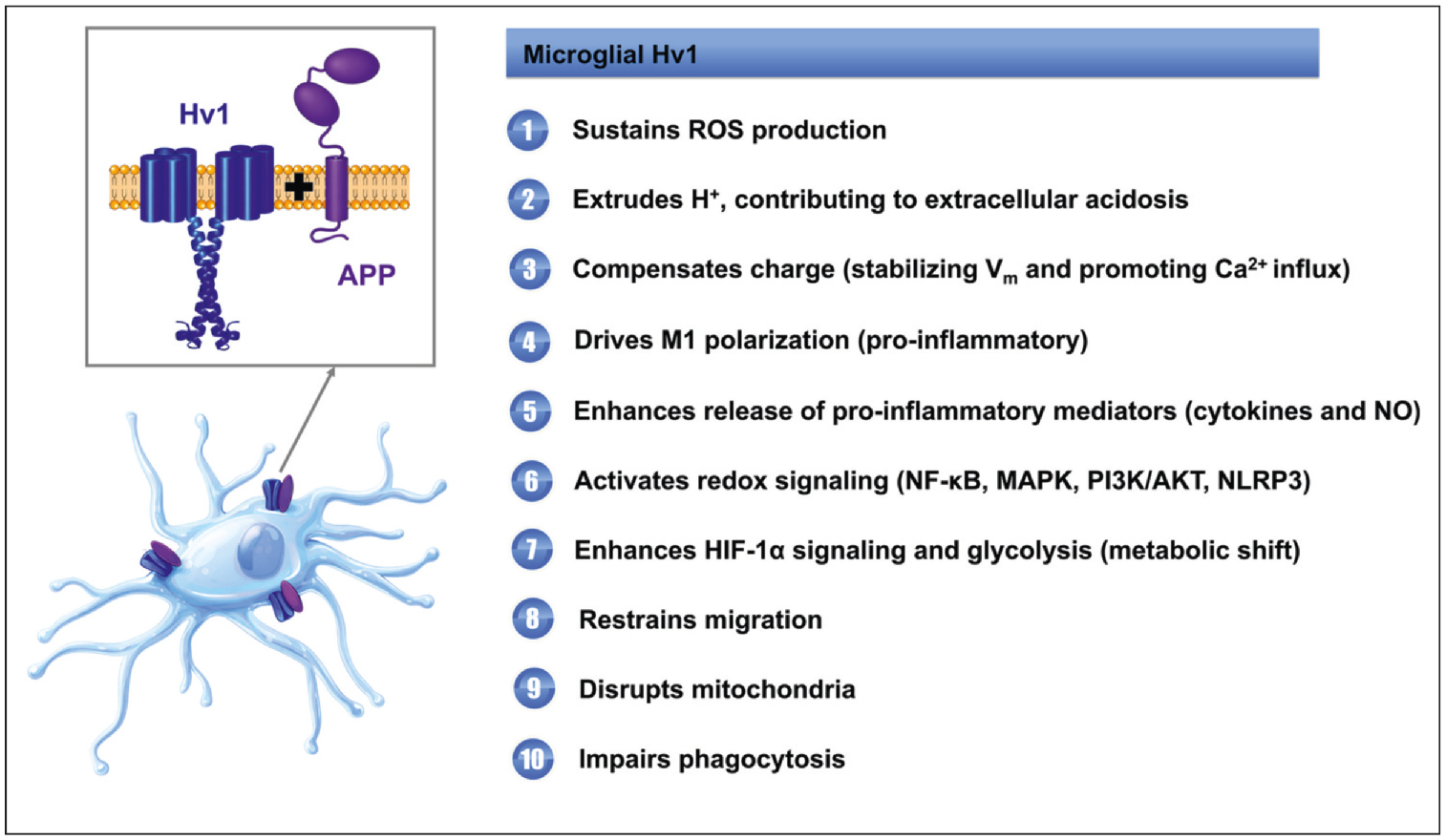
Hv1-mediated regulation of microglial functions. APP protein assembles with Hv1 channels in microglia and promotes channel activity via its C99 transmembrane fragment. This conceptual diagram illustrates the multifaceted roles of Hv1 in microglial functions. Hv1 sustains NOX-dependent ROS production, regulates intracellular pH homeostasis (while promoting extracellular acidification), stabilizes membrane potential to enhance Ca^2+^ influx, and biases microglia toward a pro-inflammatory phenotype characterized by elevated cytokines and nitric oxide release. These Hv1-driven alterations activate downstream redox signaling and HIF-1α pathways, restrict microglial migratory capacity, impair mitochondrial integrity and energy metabolism, and ultimately reduce phagocytic clearance. Collectively, these Hv1-regulated mechanisms synergistically exacerbate neuroinflammation and neuronal injury in CNS disorders. The boxed cartoon illustrates Hv1 and APP subunits in a cell membrane.

Studies using Hv1-deficient mice have implicated Hv1 channels in multiple interconnected roles in microglial physiology (Figure 1): (1) Hv1 enhances ROS generation by sustaining NOX activity [5,7,11]. (2) Hv1 extrudes protons to prevent cytosolic acidification, maintaining a near-neutral intracellular pH during the respiratory burst while acidifying the extracellular environment [6]. This extracellular acidosis can stimulate neuronal acid-sensing ion channels, exacerbating neurotoxicity [12]. (3) Hv1 stabilizes the membrane potential by providing charge compensation during depolarization, which favors hyperpolarization and increases Ca^2+^ influx [13]. (4) Hv1 promotes pro-inflammatory (M1) microglial activation, whereas loss of Hv1 biases microglia toward an anti-inflammatory (M2) phenotype [14]. (5) Hv1-driven pro-inflammatory polarization is associated with heightened production of inflammatory mediators (e.g. TNF-α, IL-1β, IL-6, and nitric oxide) [7,15]. (6) By sustaining ROS production, Hv1 enhances redox-sensitive signaling pathways, including nuclear factor kappa B (NF-κB), mitogen-activated protein kinase (MAPK), and phosphoinositide 3-kinase/protein kinase B (PI3K/AKT) signaling, as well as activation of the NOD-like receptor family pyrin domain-containing 3 (NLRP3) inflammasome, thereby promoting pro-inflammatory gene transcription [16–21]. (7) Hv1 activity is associated with enhanced hypoxia-inducible factor-1α (HIF-1α) signaling, which in turn facilitates a metabolic shift toward aerobic glycolysis, thereby supporting sustained inflammatory gene expression during microglial activation [19,22]. (8) Hv1 activity restrains microglial migration, thereby limiting microglial accumulation at sites of neuronal injury [21]. (9) Hv1 disrupts microglial mitochondrial function, leading to reduced ATP production and defective mitophagy [20]. (10) Hv1-associated mitochondrial deficits impair microglial phagocytosis, limiting efficient clearance of pathological tau aggregates during neurodegenerative conditions [20].

Through the combined actions of these mechanisms, Hv1 in microglia enhances inflammatory responses and exacerbates neuronal injury. Notably, hyperactive microglial Hv1 has been implicated in numerous inflammatory central nervous system (CNS) disorders, including ischemic stroke [23,24], spinal cord injury [25], neuropathic pain [26], multiple sclerosis [27], amyotrophic lateral sclerosis (ALS) [21], Parkinson’s disease [15], and Alzheimer’s disease (AD) [7,20]. Collectively, these observations establish Hv1 as a critical regulator of microglial activation and highlight it as a potential therapeutic target in neuroinflammatory conditions.

It is important to note that insights derived from Hv1 knockout models should be interpreted cautiously when extrapolating to humans for at least two reasons. First, Hv1 expression patterns in the mouse brain differ from those in humans. In mice, RNA sequencing analyses indicate that the HVCN1 gene is predominantly expressed in microglia, with fragments per kilobase of transcript per million mapped reads (FPKM) values ~41-fold higher than in astrocytes and 124-fold higher than in endothelial cells [28]. In contrast, in the human brain, HVCN1 expression is only modestly enriched in microglia, showing levels approximately 1.3-fold higher than in astrocytes and 1.4-fold higher than in endothelial cells [29]. Second, changes in Hv1 expression in response to inflammatory stimulation are opposite in mouse and human microglia. In primary cultures of C57BL/6J mouse microglia, lipopolysaccharide (LPS; 100 ng/mL for 6 hours) induces a statistically significant twofold increase in HVCN1 expression, while systemic LPS administration (1 mg/kg) results in an ~sixfold increase in the striatum compared with saline-treated controls [15]. In contrast, human iPSC-derived microglia (iMG) treated for 24 hours with LPS (100 ng/mL) show reduced HVCN1 expression by ~88%, and LPS administration (2 mg/kg) in mice transplanted with human iMG shows a ~68% reduction [30].

## Amyloid precursor protein is an accessory subunit in Hv1 channels

Human Hv1 channels are homodimers with two proton-selective channels, one in each subunit. The two pores gate cooperatively and are strongly influenced by the transmembrane pH gradient. In native cells, Hv1 often exhibits biophysical properties different from those observed in heterologous expression systems. For example, the half-maximal activation voltage (V_½_) that we measure for human Hv1 heterologously expressed in HEK293T cells is +28 mV using whole-cell patch-clamp with pH_o_ = 7.5 and pH_in_ = 6.0 [7]. Under identical conditions, the V_½_ of native Hv1 in human neutrophils is +42 mV, while it is +14 mV in iMG, and +15 mV in human sperm [7,31]. In other words, Hv1 opens more easily in microglia and sperm, requiring less depolarization than in HEK293T cells and neutrophils. These differences suggested to us that Hv1 might associate with tissue-specific accessory subunits that modulate voltage-dependent gating.

Indeed, while Hv1 was long thought to function as a pristine Hv1 subunit homodimer, we found that microglial Hv1 is modulated by an accessory protein: the APP [7]. APP is a transmembrane protein widely expressed in the brain (including in microglia) and is well known for its association with AD; APP is cleaved to produce a C-terminal 99-residue fragment (C99) and then smaller amyloid-β (Aβ) peptides [32,33]. Our findings indicate that APP and its C99 transmembrane fragment directly associate with Hv1 in microglia to produce the channel function observed in the native cells [7].

**Figure 2:**
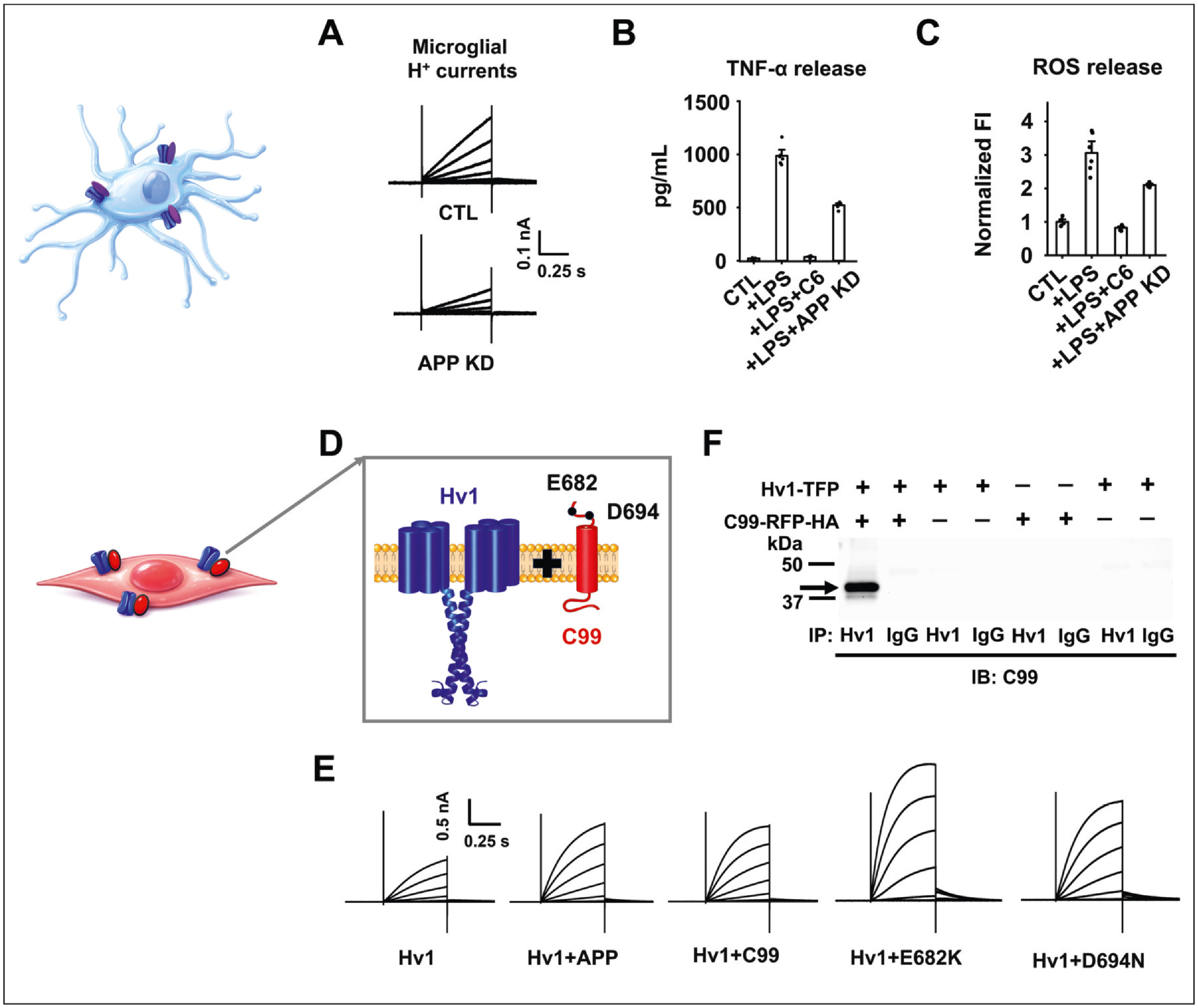
Regulation of Hv1 channels by APP and its C99 fragment. APP assembles with Hv1 in microglia. **(a)** KD of APP reduces proton currents in microglia. **(b)** The diminished Hv1 activity due to KD results in dampened release of pro-inflammatory cytokines and **(c)** production of inflammatory ROS in response to LPS; C6 peptide inhibits the response fully. **(d)** The cartoon illustrates the co-assembly of Hv1 and C99 within the cell membrane. **(e)** In HEK293T cells, co-expression of APP increases Hv1 currents by favoring channel opening at more negative membrane potentials. C99 is sufficient to assemble with Hv1 and potentiate channel function. Two early-onset AD mutations in APP (E682K and D694N) that reside within C99 significantly increase voltage-dependent channel activity beyond that induced by wild-type C99. **(f)** Co-immunoprecipitation shows that isolation of Hv1 brings along C99, demonstrating they form detergent-stable complexes in HEK293T cells. Complete data and methods are present in our publication [7].

In human iMG, we observe robust voltage-gated proton Hv1 currents blocked by the specific peptide inhibitor C6. Using RNA interference to reduce APP in iMG, we found that Hv1-mediated proton currents were diminished by 60% and the V_½_ of Hv1 activation shifted +9 mV toward the profile in HEK293T cells (Figure 2a). Thus, APP depletion made it harder for Hv1 in microglia to activate. In contrast, deleting another microglial AD-linked membrane protein (TREM2) did not affect Hv1 currents, indicating that the modulatory effect was specific to APP [7].

Consistent with these electrophysiological findings, loss of APP attenuated the respiratory burst in microglia. When stimulated with LPS, control iMG produced a robust inflammatory response characterized by high levels of ROS and cytokine release, responses nearly abolished by Hv1 blockade with the C6 peptide. APP knockdown (KD) similarly blunted this response: LPS-stimulated, APP-deficient microglia released lower levels of pro-inflammatory cytokines (e.g. an ~47% reduction in TNF-α), and ~31% less ROS than controls (Figure 2b,c). These results demonstrate that APP is required for the full Hv1-dependent inflammatory activity of microglia.

To dissect the mechanism of modulation, we reconstituted the Hv1–APP interaction in HEK293T cells. Co-expressing APP with Hv1 ~doubled the proton current amplitude and shifted V_½_ of Hv1 activation by −8 mV. Co-expression also accelerated the channel’s opening kinetics by 2.3-fold and slowed its closing by 1.3-fold, consistent with APP stabilizing Hv1 in an open conformation (Figure 2e). The isolated C99 fragment of APP produced even more significant effects compared to the full-length APP, implying that the transmembrane domain harbors the key elements needed to modulate Hv1, rather than the 671 residues in the extracellular domain (Figure 2d).

An additional consequence of APP binding is an alteration in Hv1 pharmacology. Hv1 channels assembled with APP or its C99 fragment become markedly less sensitive to the peptide inhibitor C6 [8,34]. This observation suggests that APP sterically hinders inhibitor access to Hv1 or induces a channel conformation with reduced inhibitor affinity. Moreover, APP or C99 incorporation diminishes the inhibition by the smaller, non-specific blocker Zn^2+^, which binds to extracellular histidine residues of Hv1, by ~2.8-fold and 3.5-fold, respectively [7].

Direct physical association of Hv1 and APP was confirmed by co-immunoprecipitation and total internal reflection fluorescence microscopy (TIRFm). Thus, Hv1 expressed with APP or C99 shows formation of detergent-stable complexes by co-immunoprecipitation from cell lysates (Figure 2f). Further, TIRFm shows extensive colocalization at the cell surface (Manders’ colocalization coefficient = 0.86) of fluorescently-tagged Hv1 and C99 subunits.

Notably, familial AD mutations in APP’s C99 region further modified Hv1 activity. Two pathogenic APP mutations associated with early-onset AD (the E682K Leuven mutation and the D694N Iowa mutation) enhanced Hv1 currents and produced negative shifts in V_½_ of −21 mV and −18 mV, respectively, which were larger than the −13 mV shift observed for the wild-type fragment (Figure 2e). These changes were not associated with differences in Hv1 channel surface expression, demonstrating that the mutations altered the function of the intact channel complexes. These findings are consistent with the idea that APP mutations linked to AD drive hyperactivation of microglial Hv1 to exacerbate neuroinflammation, providing a potential mechanistic link between AD-related genetic factors and immune dysfunction in the brain.

## Implications of Hv1–amyloid precursor protein interaction

Once considered only a source of neuronal amyloid, APP is now recognized as an important regulator of microglial biology. APP expression increases in microglia after CNS injury or immune activation, marking it as an acute-phase, inflammation-associated protein marker in the brain [35]. Activated microglia are suggested to be a major non-neuronal source of APP, again placing the molecule at a critical interface between innate immunity and neurodegeneration [36].

APP and its proteolytic fragments (including soluble APPα and Aβ peptides) modulate many microglial functions. Under inflammatory conditions, APP often acts as a pro-inflammatory signal. For example, secreted APPα and aggregated Aβ can activate microglia to release cytokines (such as TNF-α and IL-1β), induce nitric oxide production, and increase ROS release [37,38]. At the cell surface, APP serves as a receptor or co-receptor that triggers intracellular pathways (involving JNK/p38 MAPKs and NF-κB), thereby amplifying inflammatory gene expression [39–41]. Moreover, APP is required for microglia to mount a full response to stimuli like LPS, effectively setting the ‘gain’ of innate immune activation [42].

The discovery that APP is an Hv1-associated subunit provides a new perspective on microglial activation in health and disease. It suggests that pro-inflammatory effects attributed to APP in microglia may be mediated, at least in part, through its enhancement of Hv1 channel activity. Such a mechanism may help explain prior observations showing that genetic deletion of APP improved outcomes and reduced neuroinflammation in AD models [39]. Our work demonstrating the APP–Hv1 interaction enhances inflammatory microglial behavior that is further augmented by AD-linked APP mutations, leads us to propose that excessive proton channel activity contributes to neurodegeneration [7].

## Concluding remarks

Hv1 channels have emerged as pivotal regulators of microglial function and neuroinflammation. Their activity is tuned by a neurodegeneration-related accessory protein, APP, underscoring an unexpected level of control that governs microglial activation status. Continuing to unravel these interactions will deepen our understanding of microglial biology. The identification of APP and its C99 fragment as Hv1 accessory subunits also points toward new therapeutic opportunities. Modulation of Hv1 activity or disruption of the APP–Hv1 interaction may mitigate microglia-mediated neuronal damage in AD and other CNS disorders.

More broadly, that APP and C99 serve as Hv1 accessory subunits raises the possibility that other transmembrane proteins will assemble with Hv1 to produce native cell-specific functions. Hv1 in neutrophils or sperm, for instance, might associate with different partners to account for its unique properties in those cells compared to HEK293T cells [31]. This is reminiscent of our prior discovery that Kv channel pore-forming subunits assemble with the KCNE/MiRP family of single transmembrane proteins to yield the functional differences required to fulfill their varied roles in the heart, skeletal muscle, CNS, and stomach [43–45]. Identifying such interactions for Hv1 may prove an equally satisfying avenue for future research.

## References

[1] Sasaki M, Takagi M, Okamura Y: A voltage sensor-domain protein is a voltage-gated proton channel. Science 2006, 312:589–592. 16556803 10.1126/science.1122352

[2] Ramsey IS, Moran MM, Chong JA, Clapham DE: A voltage-gated proton-selective channel lacking the pore domain. Nature 2006, 440:1213–1216. 16554753 10.1038/nature04700 PMC4084761

[3] DeCoursey TE: Voltage-gated proton channels: molecular biology, physiology, and pathophysiology of the H(V) family. Physiol Rev 2013, 93:599–652. 23589829 10.1152/physrev.00011.2012 PMC3677779

[4] Yan L, Liu JJ, Hong L: Hv1 channel in immune cells and pharmacology. Pharmacol Res 2025, 219:107885. 40721170 10.1016/j.phrs.2025.107885 PMC12914853

[5] DeCoursey TE, Morgan D, Cherny VV: The voltage dependence of NADPH oxidase reveals why phagocytes need proton channels. Nature 2003, 422:531–534. 12673252 10.1038/nature01523

[6] Morgan D, Capasso M, Musset B, Cherny VV, Rios E, Dyer MJ, DeCoursey TE: Voltage-gated proton channels maintain pH in human neutrophils during phagocytosis. Proc Natl Acad Sci USA 2009, 106:18022–18027. 19805063 10.1073/pnas.0905565106 PMC2764923

[7.••] Zhao R, Sophanpanichkul P, Chadarevian JP, Ding Y, Dai H, Nayak M, Davtyan H, Blurton-Jones M, Goldstein SAN: Amyloid precursor protein and C99 are subunits in human microglial Hv1 channels that enhance current and inflammatory mediator release. Proc Natl Acad Sci USA 2025, 122:e2509903122. 41129234 10.1073/pnas.2509903122 PMC12582281

[8] Zhao R, Kennedy K, De Blas GA, Orta G, Pavarotti MA, Arias RJ, de la Vega-Beltran JL, Li Q, Dai H, Perozo E, : Role of human Hv1 channels in sperm capacitation and white blood cell respiratory burst established by a designed peptide inhibitor. Proc Natl Acad Sci USA 2018, 115:E11847–E11856. 30478045 10.1073/pnas.1816189115 PMC6294887

[9] Zhao R, Lopez B, Schwingshackl A, Goldstein SAN: Protection from acute lung injury by a peptide designed to inhibit the voltage-gated proton channel. iScience 2023, 26:105901. 36660473 10.1016/j.isci.2022.105901 PMC9843441

[10.•] Zhao R, Lopez B, Majumder N, Kasparian A, Hua P, Dai H, Nayak M, Zyrianova T, Taylor C, Ding Y, : C6 peptide blockade of Hv1 channels inhibits neutrophil migration into the lungs to suppress *Pseudomonas aeruginosa*-induced acute lung injury. Respir Res 2025, 26:339. 41316271 10.1186/s12931-025-03409-0 PMC12661846

[11] Ramsey IS, Ruchti E, Kaczmarek JS, Clapham DE: Hv1 proton channels are required for high-level NADPH oxidase-dependent superoxide production during the phagocyte respiratory burst. Proc Natl Acad Sci USA 2009, 106:7642–7647. 19372380 10.1073/pnas.0902761106 PMC2669790

[12] Zeng WZ, Liu DS, Liu L, She L, Wu LJ, Xu TL: Activation of acid-sensing ion channels by localized proton transient reveals their role in proton signaling. Sci Rep 2015, 5:14125. 26370138 10.1038/srep14125 PMC4569896

[13] El Chemaly A, Okochi Y, Sasaki M, Arnaudeau S, Okamura Y, Demaurex N: VSOP/Hv1 proton channels sustain calcium entry, neutrophil migration, and superoxide production by limiting cell depolarization and acidification. J Exp Med 2010, 207:129–139. 20026664 10.1084/jem.20091837 PMC2812533

[14] Tian DS, Li CY, Qin C, Murugan M, Wu LJ, Liu JL: Deficiency in the voltage-gated proton channel Hv1 increases M2 polarization of microglia and attenuates brain damage from photothrombotic ischemic stroke. J Neurochem 2016, 139:96–105. 27470181 10.1111/jnc.13751 PMC5037018

[15] Neal ML, Beier EE, Hossain MM, Boyle A, Zheng J, Kim C, Mhatre-Winters I, Wu LJ, Richardson JR: Voltage-gated proton channel Hv1 regulates neuroinflammation and dopaminergic neurodegeneration in Parkinson’s disease models. Antioxidants 2023, 12:582. 36978830 10.3390/antiox12030582 PMC10044828

[16] Sharma A, Kale NB, Yadav P, Yadav S, Ranawat M, Shinde VS, Kshatri AS: Validation of Hv(1) channel functions in BV2 microglial cells using small molecule modulators. Front Cell Neurosci 2025, 19:1624224. 40800612 10.3389/fncel.2025.1624224 PMC12340723

[17] Yu Y, Yu Z, Xie M, Wang W, Luo X: Hv1 proton channel facilitates production of ROS and pro-inflammatory cytokines in microglia and enhances oligodendrocyte progenitor cells damage from oxygen-glucose deprivation in vitro. Biochem Biophys Res Commun 2018, 498:1–8. 28676401 10.1016/j.bbrc.2017.06.197

[18] Li X, Yu Z, Zong W, Chen P, Li J, Wang M, Ding F, Xie M, Wang W, Luo X: Deficiency of the microglial Hv1 proton channel attenuates neuronal pyroptosis and inhibits inflammatory reaction after spinal cord injury. J Neuroinflamm 2020, 17:263. 10.1186/s12974-020-01942-x PMC7487532 32891159

[19.•] Sun L, Wang X, Guan S, Zhang P, Chen D, Luo T: Deficiency of microglial Hv1 protects against lipopolysaccharide-induced neuroinflammation via the NF-kappaB signaling pathway and HIF1alpha-mediated metabolic reprogramming. FASEB J 2025, 39:e70894. 40911376 10.1096/fj.202402271RRR

[20.••] Lin J, Han H, Wu K, Wu X, Shen J, Mo Y, Zhang Q, Yang H, Yu Z: Hv1 inhibition rescues AD pathology by restoring microglial mitochondrial function and enhancing mitochondrial transfer. Exp Mol Med 2025, 57:2833–2851. 41402464 10.1038/s12276-025-01593-z PMC12800102

[21.•] Wang F, Zhang KY, Zhu LJ, Li WJ, Wu Y, Gao X, Ma XR, Yin XH, Wu JB, Ye XK, : Microglial HVCN1 deficiency improves movement and survival of SOD1(G93A) ALS mice by enhancing microglial migration and neuroprotection. Adv Sci 2026,e12149. 10.1002/advs.202512149 PMC12955916 41486410

[22] Sun L, Wang X, Guan S, Chi L, Liang M, Lu X, Luo T: Inhibition of voltage-gated Hv1 alleviates LPS-induced neuroinflammation via regulation of microglial metabolic reprogramming. Int Immunopharmacol 2024, 127:111361. 38145600 10.1016/j.intimp.2023.111361

[23] Wu LJ, Wu G, Akhavan Sharif MR, Baker A, Jia Y, Fahey FH, Luo HR, Feener EP, Clapham DE: The voltage-gated proton channel Hv1 enhances brain damage from ischemic stroke. Nat Neurosci 2012, 15:565–573. 22388960 10.1038/nn.3059 PMC3314139

[24.•] Yang Z, Jin L, Li L, Wu Y, Liu W, Feng X, Li L, Jin F, Bi Y, Li R, : Brain targeted lipid nanoparticles with Hv1 inhibitors alleviate neuroinflammation post-ischemic stroke. J Nanobiotechnology 2025, 23:464. 40598588 10.1186/s12951-025-03540-6 PMC12211482

[25] Murugan M, Zheng J, Wu G, Mogilevsky R, Zheng X, Hu P, Wu J, Wu LJ: The voltage-gated proton channel Hv1 contributes to neuronal injury and motor deficits in a mouse model of spinal cord injury. Mol Brain 2020, 13:143. 33081841 10.1186/s13041-020-00682-6 PMC7574559

[26] Peng J, Yi MH, Jeong H, McEwan PP, Zheng J, Wu G, Ganatra S, Ren Y, Richardson JR, Oh SB, : The voltage-gated proton channel Hv1 promotes microglia-astrocyte communication and neuropathic pain after peripheral nerve injury. Mol Brain 2021, 14:99. 34183051 10.1186/s13041-021-00812-8 PMC8240390

[27] Chen M, Yang LL, Hu ZW, Qin C, Zhou LQ, Duan YL, Bosco DB, Wu LJ, Zhan KB, Xu SB, : Deficiency of microglial Hv1 channel is associated with activation of autophagic pathway and ROS production in LPC-induced demyelination mouse model. J Neuroinflamm 2020, 17:333. 10.1186/s12974-020-02020-y PMC7646080 33158440

[28] Zhang Y, Chen K, Sloan SA, Bennett ML, Scholze AR, O’Keeffe S, Phatnani HP, Guarnieri P, Caneda C, Ruderisch N, : An RNA-sequencing transcriptome and splicing database of glia, neurons, and vascular cells of the cerebral cortex. J Neurosci 2014, 34:11929–11947. 25186741 10.1523/JNEUROSCI.1860-14.2014 PMC4152602

[29] Zhang Y, Sloan SA, Clarke LE, Caneda C, Plaza CA, Blumenthal PD, Vogel H, Steinberg GK, Edwards MS, Li G, : Purification and characterization of progenitor and mature human astrocytes reveals transcriptional and functional differences with mouse. Neuron 2016, 89:37–53. 26687838 10.1016/j.neuron.2015.11.013 PMC4707064

[30] Hasselmann J, Coburn MA, England W, Figueroa Velez DX, Kiani Shabestari S, Tu CH, McQuade A, Kolahdouzan M, Echeverria K, Claes C, : Development of a chimeric model to study and manipulate human microglia in vivo. Neuron 2019, 103:1016–1033 e1010. 31375314 10.1016/j.neuron.2019.07.002 PMC7138101

[31] Zhao R, Dai H, Arias RJ, De Blas GA, Orta G, Pavarotti MA, Shen R, Perozo E, Mayorga LS, Darszon A, : Direct activation of the proton channel by albumin leads to human sperm capacitation and sustained release of inflammatory mediators by neutrophils. Nat Commun 2021, 12:3855. 34158477 10.1038/s41467-021-24145-1 PMC8219737

[32] Takasugi N, Komai M, Kaneshiro N, Ikeda A, Kamikubo Y, Uehara T: The pursuit of the “Inside” of the amyloid hypothesis-is C99 a promising therapeutic target for Alzheimer’s disease? Cells 2023, 12. 10.3390/cells12030454 PMC9914381 36766796

[33] O’Brien RJ, Wong PC: Amyloid precursor protein processing and Alzheimer’s disease. Annu Rev Neurosci 2011, 34:185–204. 21456963 10.1146/annurev-neuro-061010-113613 PMC3174086

[34] Zhao R, Shen R, Dai H, Perozo E, Goldstein SAN: Molecular determinants of inhibition of the human proton channel hHv1 by the designer peptide C6 and a bivalent derivative. Proc Natl Acad Sci USA 2022, 119:e2120750119. 35648818 10.1073/pnas.2120750119 PMC9191634

[35] Banati RB, Gehrmann J, Czech C, Monning U, Jones LL, Konig G, Beyreuther K, Kreutzberg GW: Early and rapid de novo synthesis of Alzheimer beta A4-amyloid precursor protein (APP) in activated microglia. Glia 1993, 9:199–210. 7507467 10.1002/glia.440090305

[36] Banati RB, Gehrmann J, Lannes-Vieira J, Wekerle H, Kreutzberg GW: Inflammatory reaction in experimental autoimmune encephalomyelitis (EAE) is accompanied by a microglial expression of the beta A4-amyloid precursor protein (APP). Glia 1995, 14:209–215. 7591032 10.1002/glia.440140306

[37] Barger SW, Harmon AD: Microglial activation by Alzheimer amyloid precursor protein and modulation by apolipoprotein E. Nature 1997, 388:878–881. 9278049 10.1038/42257

[38] Jekabsone A, Mander PK, Tickler A, Sharpe M, Brown GC: Fibrillar beta-amyloid peptide Abeta1-40 activates microglial proliferation via stimulating TNF-alpha release and H2O2 derived from NADPH oxidase: a cell culture study. J Neuroinflamm 2006, 3:24. 10.1186/1742-2094-3-24 PMC1574293 16959029

[39] Manocha GD, Floden AM, Rausch K, Kulas JA, McGregor BA, Rojanathammanee L, Puig KR, Puig KL, Karki S, Nichols MR, : APP regulates microglial phenotype in a mouse model of Alzheimer’s disease. J Neurosci 2016, 36:8471–8486. 27511018 10.1523/JNEUROSCI.4654-15.2016 PMC4978805

[40] Bodles AM, Barger SW: Secreted beta-amyloid precursor protein activates microglia via JNK and p38-MAPK. Neurobiol Aging 2005, 26:9–16. 15585341 10.1016/j.neurobiolaging.2004.02.022

[41] Guo Q, Robinson N, Mattson MP: Secreted beta-amyloid precursor protein counteracts the proapoptotic action of mutant presenilin-1 by activation of NF-kappaB and stabilization of calcium homeostasis. J Biol Chem 1998, 273:12341–12351. 9575187 10.1074/jbc.273.20.12341

[42] Carrano A, Das P: Altered innate immune and glial cell responses to inflammatory stimuli in amyloid precursor protein knockout mice. PLoS One 2015, 10:e0140210. 26447481 10.1371/journal.pone.0140210 PMC4598170

[43] Abbott GW, Sesti F, Splawski I, Buck ME, Lehmann MH, Timothy KW, Keating MT, Goldstein SA: MiRP1 forms IKr potassium channels with HERG and is associated with cardiac arrhythmia. Cell 1999, 97:175–187. 10219239 10.1016/s0092-8674(00)80728-x

[44] Abbott GW, Butler MH, Bendahhou S, Dalakas MC, Ptacek LJ, Goldstein SA: MiRP2 forms potassium channels in skeletal muscle with Kv3.4 and is associated with periodic paralysis. Cell 2001, 104:217–231. 11207363 10.1016/s0092-8674(01)00207-0

[45] Abbott GW, Goldstein SA: A superfamily of small potassium channel subunits: form and function of the MinK-related peptides (MiRPs). Q Rev Biophys 1998, 31:357–398. 10709243 10.1017/s0033583599003467

